# Parkinson’s Disease: Are PINK1 Activators Inching Closer to the Clinic?

**DOI:** 10.1021/acsmedchemlett.3c00070

**Published:** 2023-06-15

**Authors:** Youcef Mehellou

**Affiliations:** Cardiff School of Pharmacy and Pharmaceutical Sciences, Cardiff University, Redwood Building, King Edward VII Avenue, Cardiff CF10 3NB, U.K.

**Keywords:** PINK1, Activation, Mitophagy, Phosphoubiquitin, Parkinson’s disease, Neurodegeneration

## Abstract

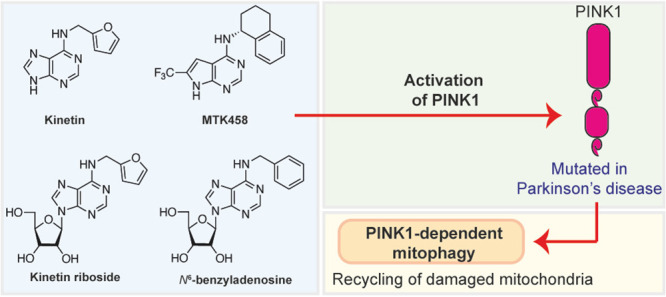

The activation of PINK1 by small molecules has emerged as a promising strategy in treating Parkinson’s disease (PD). Recent progress in this area has raised excitement around PINK1 activators as PD treatments, and herein we offer insight into these developments and their potential to deliver much needed novel PD treatments.

Mitochondrial dysfunction plays a major role in the pathogenesis of an array of neurodegenerative diseases.^1−3^ Indeed, there have been various reports linking mitochondrial dysfunction to the degeneration of dopaminergic neurons, which contribute to the motor disorder Parkinson’s disease (PD).^4^ Additionally, mitochondrial toxins associated with increased Parkinsonism, such as 1-methyl-4-phenyl-1,2,3,6-tetrahydropyridine (MPTP) and rotenone, have been shown to cause the degeneration of dopaminergic neurons in *in vivo* models.^5^ Furthermore, mitochondrial dysfunction has been linked to the generation of α-synuclein aggregates, a pathological hallmark of PD. Indeed, α-synuclein aggregates have been shown to accumulate in the mitochondria and compromise mitochondrial recycling via the autophagic machinery, a process termed mitophagy.^6,7^ Past studies have provided a direct link between mitochondrial dysfunction and PD.

Among the key enzymes involved in triggering the clearance of dysfunctional mitochondria is the mitochondrial serine/threonine PTEN-induced kinase 1 (PINK1). In 2004, it was reported that genetic mutations in PINK1 cause a familial form of PD in humans.^8^ This resulted in a growing interest in understanding the molecular mechanism by which PINK1 mutations cause PD. An early insight into this was the discovery that PD-causing PINK1 mutations cause a loss of function that compromise the kinase activity of PINK1.^9^ Other important understandings in PINK1 signaling followed, in particular the identification of ubiquitin and the E3 ubiquitin ligase, parkin, as two PINK1 physiological substrates. Notably, ubiquitin is found with α-synuclein in the proteinaceous Lewy bodies, which typify the disease, while parkin mutations cause early-onset familial forms of PD.^10,11^

Nowadays, we seem to have a good understanding of PINK1’s role in mitochondrial quality. Typically, PINK1 is inactive and is constitutively recruited to the outer mitochondrial membrane (OMM), where it undergoes proteasomal degradation in the cytosol following its *N*-terminal cleavage by proteases^12,13^ on the mitochondrial membrane (Figure 1a).^14−16^ However, when the mitochondria is damaged, PINK1 gets stabilized on the OMM in its full-length form (Figure 1b).^17−19^ This leads to the accumulation of PINK1 on the OMM, and consequently it *trans*-autophosphorylates, resulting in its activation.^20,21^ Active PINK1 then phosphorylates the E3 ubiquitin ligase parkin at serine 65^22^ and ubiquitin also at serine 65.^23^ This ultimately results in the ubiquitylation of various proteins on the OMM, resulting in mitochondrial degradation via mitophagy.^24^ With this understanding of PINK1 signaling, and the fact that PINK1 mutations that cause PD are loss-of-function mutations, it was hypothesized that the activation of PINK1, potentially through the use of small molecules, would initiate the removal of damaged neuronal mitochondria via the autophagic machinery, enabling improved neuronal survival and hence offering a new strategy for treating PD.

**Figure 1:**
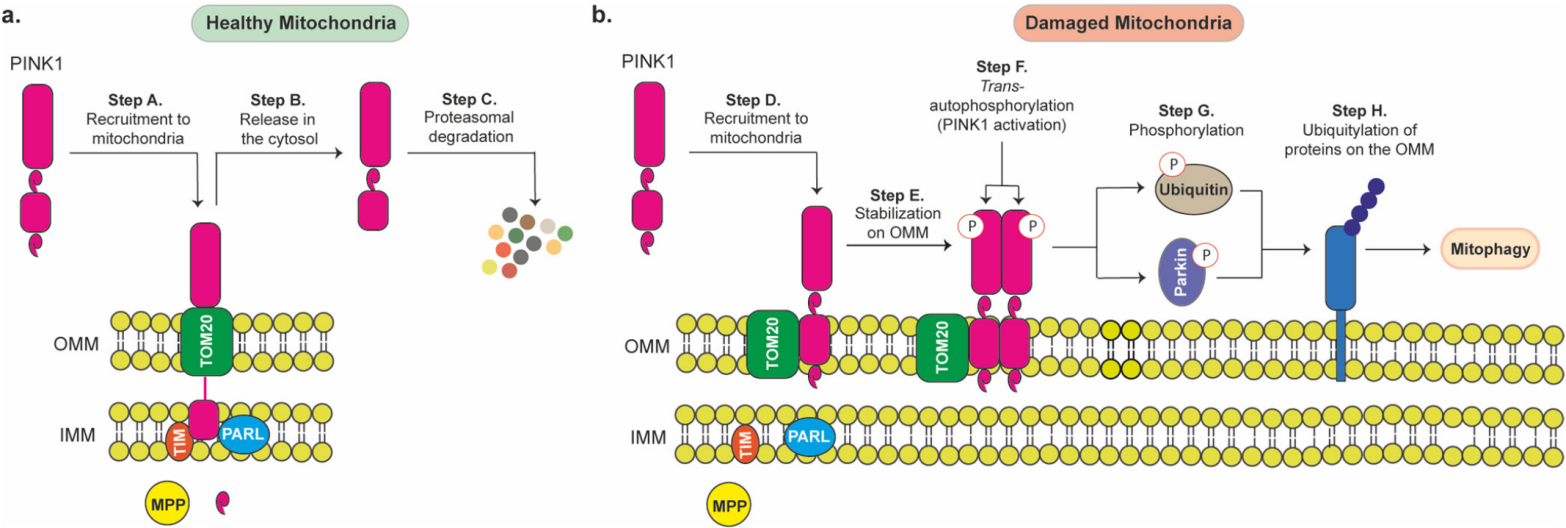
**PINK1 signaling.** PINK1 function depends on mitochondrial quality. **a**. In healthy mitochondria, PINK1 is constitutively recruited to the outer mitochondria membrane (OMM), where it undergoes N-terminal cleavage by mitochondrial proteases MPP and PARL (Step A), resulting in its release in the cytosol (Step B) and proteasomal degradation (Step C). **b**. In damaged mitochondria, PINK1 is recruited to the mitochondria (Step D), where it gets stabilized, leading to dimerization (Step E) and *trans*-autophosphorylation, resulting in its activation (Step F). Activated PINK1 directly phosphorylates (Step G) ubiquitin and parkin, leading to its activation. Active parkin ubiquitylates various proteins on the OMM (Step H), using phosphoubiquitin, resulting in mitophagy, i.e., the removal of damaged mitochondria.

To date, there have been two types of PINK1 activators reported in the literature. These are either direct PINK1 activators which bind to PINK1 directly or indirect PINK1 activators which depolarize the mitochondrial membrane potential, resulting in PINK1 activation.^25^ Since PINK1-activating mitochondrial uncouplers, i.e., indirect activators such as carbonyl cyanide *m*-chlorophenyl-hydrazine (CCCP) and niclosamide, are associated with *in vivo* toxicity and poor drug-like properties, they have not been further explored as potential therapeutics. However, this has not been the case for direct PINK1 activators.

The first discovery of a direct PINK1 activator was that of the *N*^6^-substituted adenine, kinetin (**1**, Figure 2).^26^ This compound was reported to activate PINK1 after its metabolism to the corresponding kinetin riboside triphosphate, which acts as a PINK1 ATP neosubstrate.^26^ Critically, PINK1 activation in cells by kinetin was observed when it was used with a low dose of the mitochondrial depolarizing agent CCCP. We followed this up by showing that the nucleoside derivative of kinetin, kinetin riboside (**2**, Figure 2), activates PINK1 in cells independent of mitochondrial depolarization and CCCP treatment.^27^ In the studies of kinetin and kinetin riboside activation of PINK1 in cells, the phosphorylations of the anti-apoptotic protein Bcl-xL at serine 62 and parkin at serine 65, respectively, were used as a read-out of PINK1 activity. Admittedly, both kinetin and kinetin riboside lacked potency in activating PINK1 as they were used at a relatively high concentration, 50 μM.^26,27^

**Figure 2:**
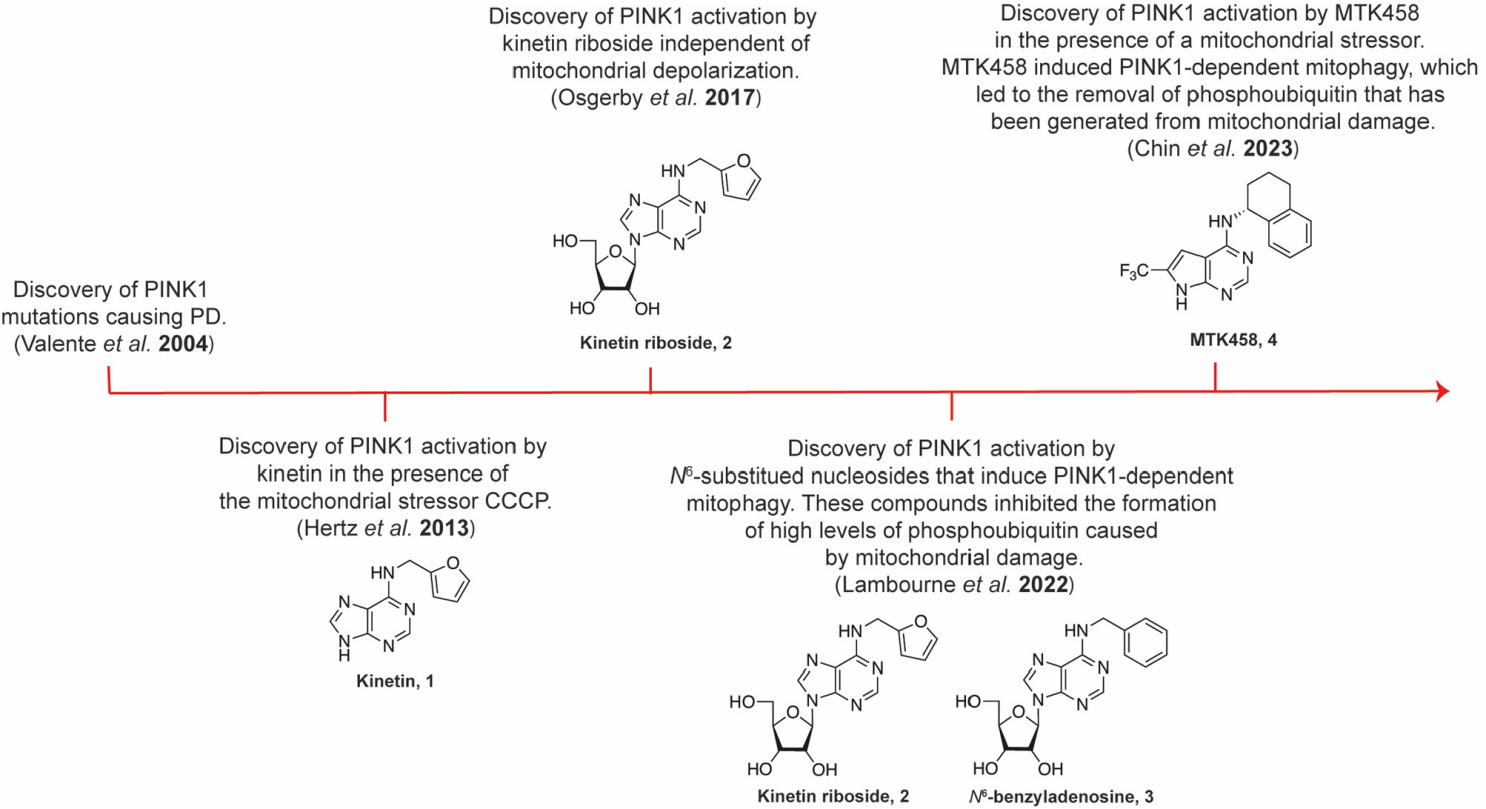
**Chemical structures of key PINK1 activators and their main biological activity.** The compounds are presented from the first report of PINK1 mutations causing PD in 2004 to the most recent PINK1 activator reported in 2023.

Although the activation of PINK1 also leads to the direct phosphorylation of ubiquitin at serine 65 to form phosphoubiquitin (Figure 1b), ubiquitin phosphorylation at serine 65 would have been considered a desirable outcome since it is an important step leading to PINK1-dependent mitophagy. Indeed, it was envisaged that the activity of small-molecule PINK1 activators could be measured by ubiquitin phosphorylation such that higher PINK1 activation would result in higher ubiquitin phosphorylation. Ultimately, this led to the notion that ubiquitin phosphorylation is desired, as it contributes to the initiation of mitophagy and the removal of damaged mitochondria in dopaminergic neurons. However, two seminal publications found that, in the brains of idiopathic PD and Lewy body dementia, there were elevated levels of phosphoubiquitin.^28,29^ Indeed, these two studies alluded to the fact that significant accumulation of phosphoubiquitin may not be desirable in treating PD and Lewy body dementia, and rather may contribute to the pathology of these neurological disorders. Recent discoveries around PINK1 activation have since answered these dilemmas, as discussed below.

Working on discovering potent PINK1 activators, our lab examined the combination of the PINK1 activator kinetin riboside and other related *N*^6^-substituted adenosines (**2** and **3**, Figure 2) with the mitochondrial uncoupler CCCP as potential strategy for achieving maximal PINK1 activation.^30,31^ These nucleoside analogues caused PINK1 activation in cells, as judged by the phosphorylation of parkin, the physiological substrate of PINK1. Surprisingly, pretreatment with these compounds inhibited the elevated CCCP- and niclosamide-induced ubiquitin phosphorylation. Further, these nucleoside analogues were able to cause low-level PINK1-dependent mitophagy and limited the localization of phosphoubiquitin to the mitochondria. Hence, these compounds caused the desired PINK1-dependent mitophagy while they inhibited formation of high levels of phosphoubiquitin, a hallmark of PD. Although these studies were initially conducted in HeLa cells overexpressing parkin, we subsequently showed that these compounds inhibited the endogenous ubiquitin phosphorylation at serine 65 in astrocytes that is caused by the mitochondrial uncoupler valinomycin. Notably, the activation of PINK1 and inhibition of CCCP-mediated ubiquitin phosphorylation by the nucleoside analogues were not observed with the prodrugs of the monophosphate derivatives of these nucleosides. This suggested that the further phosphorylation of the nucleosides into their triphosphate species was not required for PINK1 activation. Nevertheless, our discovery was the first literature report showing (1) the ability of small-molecule PINK1 activators to limit the accumulation of high levels of phosphoubiquitin that is caused by mitochondrial damaging agents and (2) their inhibition of phosphoubiquitin localization to the mitochondria, though we did not fully understand the mechanism of action of these compounds.

Our work was followed by a publication from Chin et al.^32^ where they showed that an *N*^6^-substituted adenine, called MTK458 (**4**, Figure 2), activated PINK1 and cleared phosphoubiquitin that had been generated from stalled mitophagy.^32^ It must be noted that MTK458 activation of PINK1 was observed in the presence of a low dose (0.5–1 μM) of mitochondrial stressor, e.g., carbonyl cyanide *p*-(trifluoromethoxy)phenyl-hydrazone (FCCP) and oligomycin. Critically, this work showed that MTK458 binds PINK1 directly and stabilizes it in an active conformation. Indeed, PINK1 activation was found to be caused directly by MTK458 and not its metabolite(s), the nucleoside derivative or its phosphorylated species, e.g., the triphosphate derivative, unlike how the *N*^6^-substituted adenine, kinetin **1**, was reported previously to activate PINK1.^26^ This study went further and showed that mitochondrial damage caused by mitochondrial depolarizing agents, such as CCCP, leads to the formation of phosphoubiquitin, but due to impaired mitophagy, phosphoubiquitin was not further processed and accumulated in cells. This impaired mitophagy was corrected by the PINK1 activator, MTK458, which triggered PINK1-dependent mitophagy. This in turn resulted in the removal of the accumulated phosphoubiquitin. Indeed, MTK458 was found to clear mitochondrial aggregates via PINK1-dependent mitophagy in cells and decreased ubiquitin phosphorylation in the brain and plasma from PD animal models. Furthermore, daily oral dosing of MTK458 in an α-synuclein PD mouse model led to a dose-dependent decrease in α-synuclein pathology in 3-month and 7-month studies. This was accompanied by a rescue of free movement and motor activity along with a reduction in inflammatory markers.

The studies by Lambourne et al.^30,31^ and Chin et al.^32^ have brought us to the understanding that small molecules that activate PINK1 and lead to PINK1-dependent mitophagy, which suppress the accumulation of high levels of phosphoubiquitin, are feasible. Structurally, both groups’ studies alluded to the fact that highly phosphorylated metabolites of the nucleobase- and nucleoside-based compounds, e.g., their triphosphate derivatives, are not needed for the activation of PINK1. This will have great implications in the future design of PINK1 activators, as glycosylation of *N*^6^-substituted adenines and nucleobases, and their phosphorylation to their triphosphate derivatives, are not required for achieving PINK1 activation. This was beautifully illustrated by Chin et al.,^32^ where MTK458 (**4**, Figure 2) acted directly on PINK1 without the need for glycosylation and phosphorylation. Indeed, this allowed for the design of brain-penetrant PINK1 activators with good oral bioavailability, an essential criterion for molecules that can treat PD. Critically, this study addressed an outstanding question in the field of discovering PINK1 activators as treatments for PD: the identification of suitable biomarkers for measuring PINK1 activity in individuals. Chin et al.^32^ showed that levels of phosphoubiquitin in plasma correlate with the progression of PD, and this could be used as a read-out of PINK1 activity in humans. Together, these findings provided significant de-risking of the pharmacological activation PINK1 to treat PD.

Overall, there is no doubt that, since the discovery of PINK1 mutations causing PD in 2004, the field has made great progress in understanding the molecular function of PINK1 within the context of PD pathology. Recent discoveries of PINK1 activators provided a useful blueprint for the future discovery of small-molecule PINK1 activators that could treat PD by triggering PINK1-dependent mitophagy and clearing accumulated phosphoubiquitin that results from impaired mitophagy. With these drug design insights and the validation of a biomarker for PINK1 activity *in vivo*, the path for developing PINK1 activators as treatments for PD is now clearer than ever before. To maximize the chances of realizing a PINK1 activator as a clinically used treatment for PD, and potentially other neurodegenerative diseases, further efforts are needed to identify various small-molecule PINK1-activating clinical candidates to circumvent potential future setbacks in clinical trials regarding pharmacokinetics, pharmacodynamics, and toxicity profiles that may be associated with one or more of these clinical candidates.

Together, the recent progress made in discovering PINK1 activators can only be viewed as a significant step forward in the validation of PINK1 activation, by small molecules, as a promising strategy for developing a new class of PD treatments, which PD patients desperately need.

## Abbreviations

CCCP: carbonyl cyanide *m*-chlorophenyl-hydrazine
FCCP: carbonyl cyanide *p*-(trifluoromethoxy)phenyl-hydrazone
OMM: outer mitochondrial membrane
PD: Parkinson’s disease
PINK1: PTEN-induced kinase 1
